# Anshen Jieyu Decoction: A Promising Remedy for Depression via AKT/mTOR Pathway Modulation in CUMS Rats

**DOI:** 10.1002/brb3.71380

**Published:** 2026-03-31

**Authors:** Zhen Li, Zijun Ji, Zehao Zhang, Xuzhang Wang, Hongyue Yu, Dongrun Yang, Huijin Zhang, Yongsheng Liu, Zhuxin Sui

**Affiliations:** ^1^ Undergraduate Academic Affairs Office Qilu Medical University Zibo China; ^2^ College of Medical Imaging Qilu Medical University Zibo China; ^3^ College of Clinical Medicine Qilu Medical University Zibo China; ^4^ School of Public Health Qilu Medical University Zibo China; ^5^ School of Basic Medical Sciences Qilu Medical University Zibo China

**Keywords:** AKT/mTOR signaling pathway, An Shen Jieyu decoction, depression, hypothalamic‐pituitary‐adrenal axis (HPA), rat

## Abstract

**Objective:**

To investigate the effects of An Shen Jie Yu Tang (ASJYD) on hippocampal morphology, AKT/mTOR phosphorylation, and serum adrenocorticotropic hormone (ACTH) and corticosterone (CORT) levels in rats subjected to chronic unpredictable mild stress (CUMS).

**Methods:**

Three groups were used: Control, CUMS, and CUMS+ASJYD. Over 30 days, the CUMS and CUMS+ASJYD groups were exposed to stressors. After stress induction and behavioral assessments, the CUMS+ASJYD group received ASJYD for 14 days. Serum ACTH and CORT were quantified by ELISA. Hippocampal p‐AKT/p‐mTOR protein expression was evaluated by Western blot. Hippocampal neuron morphology was examined via Nissl staining, and mitochondrial ultrastructure via transmission electron microscopy.

**Results:**

Compared to controls, CUMS rats showed reduced sucrose preference, decreased distance/time in the open field center, and increased water maze latency (*p*<0.01). ASJYD ameliorated these impairments (*p*<0.01). CUMS rats exhibited elevated ACTH/CORT and decreased p‐AKT/p‐mTOR (*p*<0.01). ASJYD reduced ACTH/CORT and increased p‐AKT/p‐mTOR (*p*<0.01). Histology revealed CUMS rats had disorganized hippocampal neurons, reduced cell numbers, and nuclear abnormalities, which were improved by ASJYD.

**Conclusions:**

ASJYD ameliorates CUMS‐induced behavioral deficits, reduces hypothalamic‐pituitary‐adrenal axis hyperactivity, is associated with increased phosphorylation of AKT and mTOR, and protects against hippocampal neuronal damage.

## Graphical abstract

An Shen Jie Yu Tang ameliorates depression‐like behaviors in chronically stressed rats. Its mechanism involves suppressing HPA axis hyperactivity and activating the hippocampal AKT/mTOR signaling pathway, leading to neuroprotective effects.

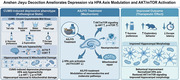

## 1 Introduction

Depression is a prevalent mental disorder that can manifest at any stage of life, characterized by persistent and recurrent episodes of emotional distress. Individuals suffering from depression frequently display a low mood and pessimistic, negative attitudes, with severe cases potentially resulting in adverse outcomes such as suicide (Liu et al. 2023). In the aftermath of the COVID‐19 pandemic, the incidence of global mental health disorders has deteriorated, with an increase of 53 million new cases of depression (Daly and Robinson 2022; “Global prevalence and burden of depressive and anxiety disorders in 204 countries and territories in 2020 due to the COVID‐19 pandemic,” 2021). According to the WHO, depression remains a major global public health problem affecting hundreds of millions of people worldwide (World Health Organization 2025). World Health Organization policy documents have projected that, by 2030, depression may become the leading contributor to global disease burden (World Health Organization 2012). The pathogenesis of depression is intricate, involving social‐psychological, neurological, and genetic factors, underscoring the growing importance of research into its mechanisms and prevention strategies (Baghaei et al. 2021; Wang et al. 2024).

The hypothalamic‐pituitary‐adrenal (HPA) axis functions as the central component of the neuroendocrine system. Prolonged stress exposure results in HPA axis hyperactivity, which disrupts the limbic system and causes damage to the hippocampus, a region essential for learning and memory. Mitochondria, recognized as the cellular powerhouses, have been implicated in various neurological disorders due to mitochondrial dysfunction (Maghsoudi et al. 2014). Symptoms of depression, including fatigue, lack of energy, difficulty concentrating, and low mood, may be linked to decreased ATP energy efficiency. Animal models of depression induced by chronic unpredictable mild stress (CUMS) have demonstrated mitochondrial structural damage, impaired ATP production, and an increase in free radical generation, contributing to brain damage (Chen, Shi, et al. 2024). These processes can trigger apoptosis of hippocampal neurons, thereby facilitating the onset of depression. The AKT/mTOR signaling pathway plays a crucial role in regulating cell growth and apoptosis, enhancing mitochondrial function, and modulating synaptic plasticity, and it is associated with various neurological disorders (Li et al. 2023). Phosphatidylinositol 3‐kinase (PI3K) mediates an antiapoptotic effect through the activation of protein kinase B (AKT) and the subsequent regulation of its downstream target, mTOR, which contributes to the alleviation of depressive symptoms and the enhancement of memory and cognitive functions (Guo et al. 2024). Collectively, these findings suggest that chronic stress–induced HPA axis hyperactivation may contribute to mitochondrial dysfunction and downstream alterations in PI3K/AKT/mTOR signaling, thereby potentially linking neuroendocrine dysregulation with impaired neuroplasticity in depression. In the framework of Traditional Chinese Medicine, depression is always attributed to liver qi stagnation with concomitant disturbance of the heart spirit. Accordingly, An Shen Jie Yu Tang Decoction (ASJYD) is formulated to “soothe the liver and relieve constraint” and “calm the mind,” aiming to improve stress‐related affective and sleep symptoms. A plausible modern rationale links ASJYD to AKT/mTOR‐related neuroplasticity: Bupleurum (Chaihu), a key component of ASJYD, contains Saikosaponin D, which alleviates depressive‐like behaviors in CUMS models while modulating hippocampal mTOR signaling and synaptic protein expression (Liu et al. 2022). Given the established involvement of PI3K/AKT/mTOR signaling in depression and antidepressant responses, we hypothesized that ASJYD may exert neuroprotective and antidepressant effects partly through regulation of AKT and mTOR phosphorylation (Chen et al. 2024).

This study investigated alterations in the expression patterns of hippocampal neurons and their mitochondria, the HPA axis, and the AKT/mTOR signaling pathway under stress‐induced depressive‐like conditions. The aim was to explore the molecular mechanisms underlying depression and to assess the effects of the ASJYD. The findings contribute to a deeper understanding of the pathophysiology of depression and its potential therapeutic interventions.

## 2 Materials

### 2.1 Main Reagents and Medicines

An Shen Jie Yu Tang (Bupleurum 10 g, Codonopsis 15 g, Poria 15 g, Atractylodes 10 g, Astragalus 10 g, Glycyrrhiza 10 g, Angelica 10 g, Longan 10 g, Ziziphus 10 g, Polygala 15 g, Aquilaria 10 g, Shen Qu 15 g, Poria cocos 10 g). The herbs for this formula were purchased from the Zibo Collaborative Hospital of Beijing Traditional Chinese Medicine Hospital. After decoction, the solution was concentrated in a 60°C water bath to a concentration of 2 g/mL of crude drug. Detailed information for ELISA kits and antibodies used in this study (supplier, catalog number, host species, and working dilution) is provided in Table 1. Major equipment included a tissue dehydrator/embedding system and paraffin microtome (Leica, Germany), a Multiskan FC microplate reader (Thermo Fisher Scientific, USA), a TD5D centrifuge (Henan Beihong, China), an electrophoresis system (Beijing Junyi Dongfang, China), and a chemiluminescence imaging system (Beijing Yuanpinghao, China).

**TABLE 1:** Primary antibodies and reagents.

Item	Application	Supplier	Catalog No.	Host	Type	Dilution/notes
**Primary antibody**	β‐actin	Hangzhou Hua'an Bio‐Technology Co., Ltd.	HA722023	Rabbit antirat	Monoclonal	1:50,000/(WB loading control)
**Primary antibody**	p‐AKT	Hangzhou Hua'an Bio‐Technology Co., Ltd.	ET1607‐73	Rabbit antirat	Monoclonal	1:5000
**Primary antibody**	p‐mTOR	Hangzhou Hua'an Bio‐Technology Co., Ltd.	ET1608‐5	Rabbit antirat	Monoclonal	1:5000
**Secondary antibody**	HRP	Hangzhou Hua'an Bio‐Technology Co., Ltd.	HA1001	Goat antirabbit	Polyclonal	1:50,000
**ELISA kit**	ACTH (serum)	Wuhan Huamei Bio‐Technology Co., Ltd.	CSB‐E06875r	——	Kit	Per manufacturer's protocol
**ELISA kit**	CORT (serum)	Wuhan Huamei Bio‐Technology Co., Ltd.	CSB‐E07014r	——	Kit	Per manufacturer's protocol

### 2.2 Experimental Animals

Purchased from Jinan Pengyue Experimental Animal Breeding Co., Ltd., 30 SPF‐grade male SD rats weighing (250 ± 20) g, license number: SCXK (Lu) 20220006, housed in the animal facility of Qilu Medical University (22–26°C) for 7 days of acclimatization; Experimental animal feed, license number: SCXK (Lu) 20230002. This experiment was approved by the Ethics Committee of Qilu Medical University, with approval number YXLL2019001.

## 3 Methods

### 3.1 Animal Experiments and Experimental Design

Twenty male Sprague‐Dawley rats (250 ± 20) g were used for CUMS modeling, with an additional 10 rats as controls. After acclimatization, rats were randomly assigned to three groups (*n* = 10 each): Control, CUMS, and CUMS+ASJYD treatment. Animal cages were rotated weekly, and all behavioral testing was conducted in a randomized order. While personnel performing CUMS were unblinded, all outcome assessments (including histological evaluations of Nissl‐stained sections) and data analysis were performed blinded. Predefined humane endpoints (e.g., >20% weight loss, persistent anorexia) and exclusion criteria were established, but no animals were excluded as all completed the study successfully. The primary outcome was the sucrose preference test (SPT) for anhedonia. Secondary outcomes were the open field test (OFT) and SPT. After behavioral testing, deep anesthesia was induced with intraperitoneal urethane. Absence of pedal and corneal reflexes confirmed anesthesia, followed by euthanasia via cardiac perfusion, with cessation of heartbeat and respiration as the death criterion. No animals survived the experiment.

### 3.2 Animal Grouping and Modeling

After 30 rats were acclimatized for 1 week, they were randomly divided into three groups of 10 rats each: the control group (Control), the CUMS group (CUMS), and the ASJYD treatment group (CUMS+ASJYD). The Control group was normally housed without any special treatment. Rats in the CUMS group and ASJYD treatment group were subjected to 10 types of CUMS over 30 days. These included: 24‐h fasting, 24‐h water deprivation, 2‐h behavioral restraint stress, 5‐min forced swimming, 24‐h strobe light exposure, 24‐h ultrasonic rodent repellent exposure, 24‐h 45° cage tilt, 1‐min tail pinching, 15‐min cage shaking, and 24‐h exposure to damp bedding. The above stimuli were repeated three times, with the order randomized but the same stressor not appearing consecutively, to prevent the rats from anticipating the stimuli (Table 2).

**TABLE 2:** CUMS modeling process.

Dates	Stimuli	Dates	Stimuli	Dates	Stimuli
7.1	24‐h food deprivation	7.11	24‐h damp bedding exposure	7.21	24‐h behavioral restraint stress
7.2	24‐h damp bedding exposure	7.12	Forced swimming	7.22	24‐h ultrasonic frequency exposure
7.3	2‐min tail pressure application	7.13	15‐min horizontal cage agitation	7.23	24‐h damp bedding exposure
7.4	45°tilted cages	7.14	24‐h ultrasonic frequency exposure	7.24	Forced swimming
7.5	24‐h behavioral restraint stress	7.15	45°tilted cages	7.25	2‐min tail pressure application
7.6	Forced swimming	7.16	24‐h continuous photic stimulation	7.26	24‐h water deprivation
7.7	24‐h water deprivation	7.17	24‐h behavioral restraint stress	7.27	15‐min horizontal cage agitation
7.8	24‐h ultrasonic frequency exposure	7.18	2‐min tail pressure application	7.28	45°tilted cages
7.9	24‐h continuous photic stimulation	7.19	24‐h water deprivation	7.29	24‐h food deprivation
7.10	15‐min horizontal cage agitation	7.20	24‐h food deprivation	7.30	24‐h continuous photic stimulation

### 3.3 Drug Intervention

After successful CUMS modeling, drug treatment was administered. The control group (Control) had free access to food and water without stimulation; the CUMS group (CUMS) ceased stimulation, had free access to food and water, and received the same volume of physiological saline via gavage as the ASJYD treatment group (CUMS+ASJYD); The CUMS+ASJYD group had free access to food and water, was administered the ASJYD daily, twice daily, at a dose of 2 mL per administration, for 14 consecutive days. During the 14‐day treatment phase, no additional CUMS stressors were applied; thus, the experimental design represents post‐stress intervention rather than treatment under ongoing stress exposure.

#### 3.3.1 Behavioral Tests (3.4−3.6)

Behavioral tests were performed at Day 1 (baseline), Day 31 (post‐modeling), and Day 45 (post‐treatment). Behavioral results presented and statistically analyzed in this manuscript were based on the Day 45 endpoint (post‐treatment) for between‐group comparisons.

### 3.4 Sugar Water Preference Test

Three SPTs were performed on Day 1 (baseline), Day 31 (post‐modeling), and Day 45 (post‐treatment). Before each test, rats were deprived of food and water for 24 h and then housed individually. Each cage was provided with one bottle of tap water (200 mL) and one bottle of 2% sucrose solution (200 mL). After 2 h, bottles were removed and weighed to calculate fluid intake. Sucrose preference was calculated as: sucrose intake / (sucrose intake + water intake) × 100%.

### 3.5 Open Field Test

OFTs were conducted on Days 1, 31, and 45. Rats were placed in the center of an open‐field arena (118 cm × 118 cm × 48 cm) and recorded for 5 min. The arena was cleaned with 75% ethanol and allowed to dry between animals. Total distance traveled and time spent in the center zone were analyzed using the Rat Doctor system. For zone‐based assessment, the arena was virtually divided using predefined settings: the center zone was defined as the central 25% of the total arena area, and the remaining area was defined as the periphery; identical zone settings were applied to all animals. To control lighting conditions, all tests were performed during a fixed time window (19:00–21:00) with the room lights turned off.

### 3.6 Morris Water Maze Experiment

The Morris water maze test was conducted at three stages (Day 1, Day 31, and Day 45). At each stage, rats underwent an acquisition protocol consisting of four trials per day for 4 consecutive days with a hidden platform. The circular platform was submerged approximately 1–2 cm below the water surface and remained invisible to the animals. In each trial, a rat was released from a designated start point facing the pool wall and allowed 60 s to locate the platform; if unsuccessful, it was guided to the platform and allowed to remain there for 10 s. Escape latency (time to reach the platform) was recorded and averaged for each rat. A probe trial was not conducted in the present study, as the primary outcome measure was escaping latency during acquisition training. Day 1 and Day 31 testing served to verify baseline comparability and successful CUMS induction.

### 3.7 ELISA

Immediately after completion of behavioral testing on Day 45 and prior to cardiac perfusion, approximately 2 mL of blood was collected from the retro‐orbital venous plexus under deep anesthesia. Blood samples were allowed to clot at room temperature and were subsequently centrifuged at 3000 rpm for 15 min to obtain serum. The serum supernatant was aliquoted and stored at −20°C for short‐term preservation until ELISA analysis, consistent with established preanalytical recommendations for serum storage (Grankvist et al. 2018). All samples were analyzed within 4 weeks of collection and were subjected to a single freeze–thaw cycle to minimize degradation. ELISA measurements were performed in duplicate according to the manufacturer's protocol to ensure analytical reliability.

### 3.8 Western Blot

Hippocampal tissues from the three experimental groups (Control, CUMS, and CUMS+ASJYD; *n* = 3 per group) were individually homogenized in lysis buffer on ice and centrifuged at 12,000 rpm for 10 min at 4°C, after which the supernatants were collected. Protein concentrations were determined using a BCA assay kit. Equal amounts of protein (40 µg per lane) were separated by SDS‐PAGE and transferred onto PVDF membranes using a wet transfer system. Membranes were blocked with 5% skim milk and incubated overnight at 4°C with primary antibodies against β‐actin, p‐AKT, and p‐mTOR, followed by incubation with HRP‐conjugated secondary antibody. Detailed antibody information, including supplier, catalog number, host species, clone type, and working dilution, is provided in Table 1. Protein bands were visualized using enhanced chemiluminescence reagents, and band intensities were quantified using ImageJ software. Target protein expression levels were normalized to β‐actin. Western blot analyses were performed using three independent biological replicates.

### 3.9 Nissl Staining

Three rats were selected from each group (*n* = 3 per group): control (Control), CUMS (CUMS), and ASJYD treatment (CUMS+ASJYD). After anesthesia, perform whole‐body blood exchange and fixation. Place brain tissue into a 4°C prechilled phosphate‐buffered solution containing 4% paraformaldehyde for washing, then place it into embedding cassettes for graded dehydration, clearing, wax immersion, and embedding. Prepare paraffin blocks and complete hippocampal tissue sectioning. Nissl staining: After dewaxing and washing with water, the tissue was stained with Nissl stain for 2–5 min, washed with water, differentiated, baked at 65°C for 4 h, and mounted with clear mount. The structural changes in the neurons of the CA1 region of the rat hippocampus were observed. Nissl staining was used for qualitative assessment of hippocampal CA1 morphology, and no stereological or quantitative neuron counting was performed.

### 3.10 Transmission Electron Microscopy Observation

Four rats were selected from each group for transmission electron microscopy (TEM) analysis (*n* = 4 per group). Rat brain tissue was immediately placed in prechilled 2.5% glutaraldehyde for fixation, stored at 4°C for 3 h, then dehydrated, osmotized, embedded, sectioned, and stained. TEM was used to observe the ultrastructure of rat hippocampal neurons and mitochondria. TEM analysis was descriptive and focused on representative ultrastructural features rather than quantitative morphometric analysis.

### 3.11 Statistical Analysis

Data statistical analysis was performed using SPSS 26.0 software. Experimental data were presented as “mean ± standard deviation (±s).” Intergroup comparisons were performed using one‐way analysis of variance (ANOVA). Differences were considered statistically significant at *p* < 0.05. Data bar charts were generated using GraphPad Prism 9.0 software, and band analysis was performed using Image J. Behavioral results (SPT, OFT, and MWM (Morris water maze)) were analyzed as endpoint measurements at Day 45 using one‐way ANOVA followed by post hoc multiple‐comparison tests.

## 4 Results

### 4.1 An Shen Jie Yu Tang Significantly Restored Rats’ Pleasurable Experiences

**FIGURE 1:**
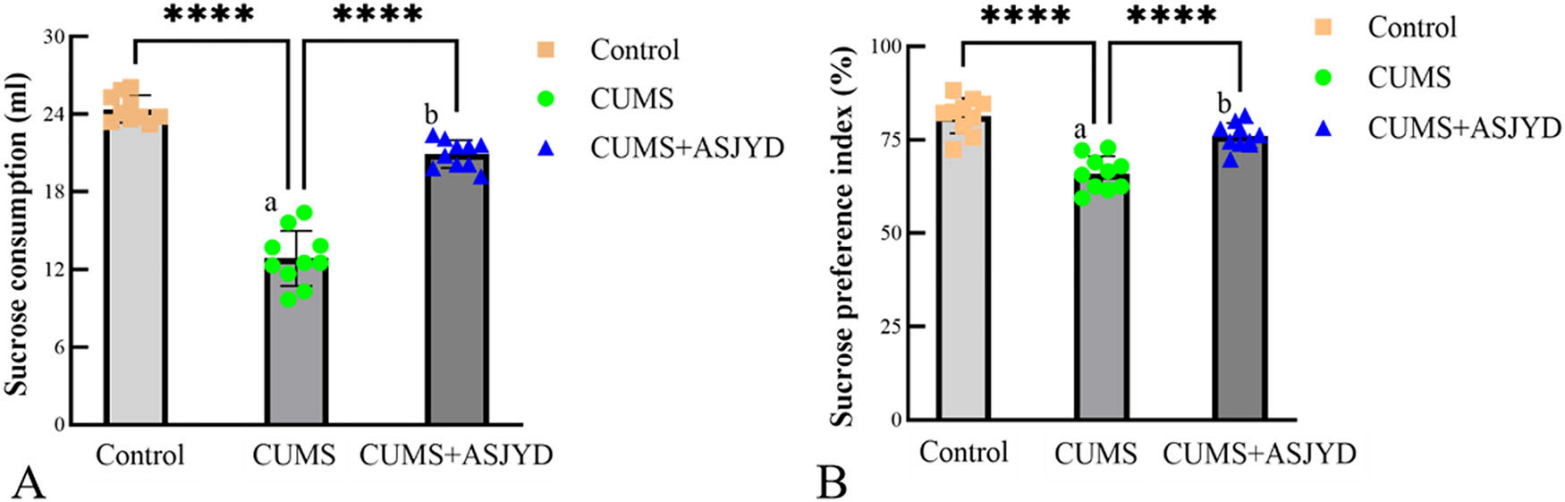
Comparison of sucrose preference between groups at Day 45 post‐treatment endpoint. (A) Sucrose consumption; (B) sucrose preference rate. ^a^ *p*<0.01 versus Control. ^b^ *p*<0.01 versus CUMS. *n* = 10 per group.

At the Day 45 post‐treatment endpoint, the sucrose consumption and sucrose preference rate were analyzed among groups. Compared with the Control group, the CUMS group showed a significant reduction in both indices (*p* < 0.01). After 2 weeks of ASJYD treatment, both indices in the CUMS+ASJYD group were significantly increased compared with the CUMS group (*p* < 0.01; Figure 1A,B), indicating that ASJYD alleviated CUMS‐induced anhedonia (A: sucrose consumption; B: sucrose preference rate).

### 4.2 The An Shen Jie Yu Tang Significantly Improved the Exploratory Interest and Motor Ability of Depressed Rats

**FIGURE 2:**
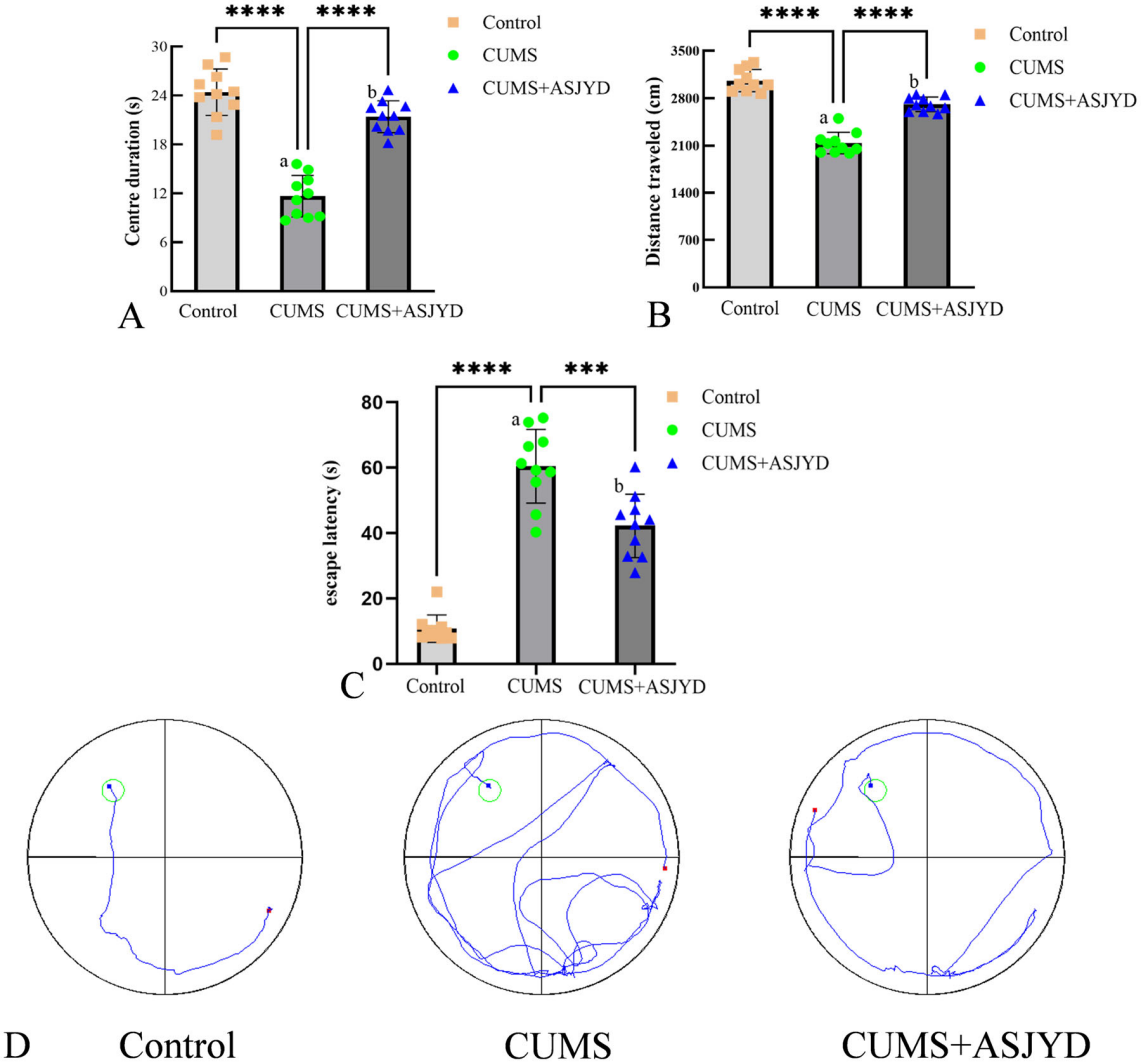
Comparison of behavioral outcomes between groups at the Day 45 post‐treatment endpoint. (A) Center duration in the open field test. (B) Distance traveled in the open field test. (C) Mean escape latency in the Morris water maze test. (D) Representative swimming trajectories in the Morris water maze test. ^a^ *p* < 0.01 versus Control; ^b^ *p* < 0.01 versus CUMS. *n* = 10 per group.

The data on central activity time and walking distance in the OFT, as well as escape latency in the Morris water maze test, were analyzed across groups. Before modeling, no statistically significant differences were observed among the three groups (*p* > 0.05). After model establishment, both the CUMS group and the ASJYD treatment group (CUMS + ASJYD) showed significantly lower locomotor activity in the OFT and significantly longer escape latencies in the Morris water maze test compared to the Control group (*p* < 0.01), indicating successful modeling. Following drug administration, the ASJYD treatment group exhibited a significant reversal of these changes, demonstrating increased locomotor and exploratory activity alongside improved spatial learning and memory compared to the CUMS group (*p* < 0.01; Figure 2A–C). These results indicate that ASJYD ameliorated depressive‐like behaviors and improved cognitive‐motor performance in rats (Figure 2D).

### 4.3 The An Shen Jie Yu Tang Can Reduce the Expression of Serum Adrenocorticotropic Hormone and Serum Corticosterone in Depressed Rats

**FIGURE 3:**
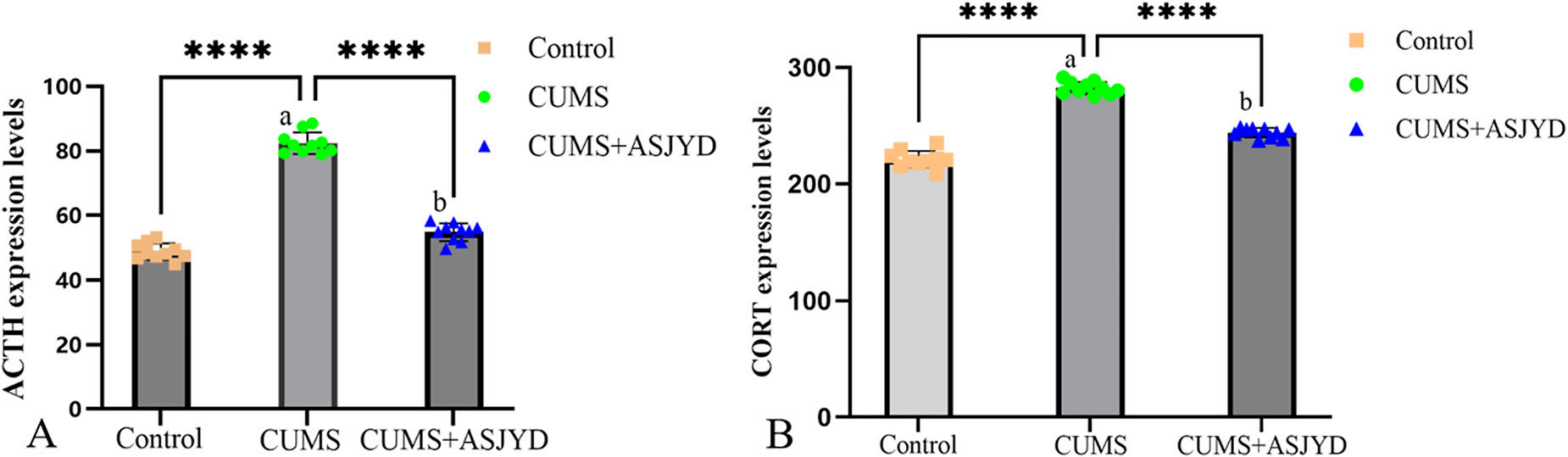
Comparison of serum ACTH and serum CORT expression levels in each group of rats after drug administration. (A) Serum ACTH expression level; (B) serum CORT expression level. ^a^ *p*<0.01 versus Control. ^b^ *p*<0.01 versus CUMS.

Compared with the Control group, serum adrenocorticotropic hormone (ACTH) and corticosterone (CORT) levels in the CUMS group were significantly elevated (*p* < 0.01; Figure 3A,B), indicating HPA axis hyperactivity under chronic stress. Compared with the CUMS group, serum ACTH and CORT levels in the CUMS+ASJYD group were significantly reduced (*p* < 0.01; Figure 3A,B), suggesting that ASJYD attenuates stress‐induced elevations in ACTH and CORT.

### 4.4 An Shen Jie Yu Tang Increases the Expression of Phosphorylated AKT and mTOR in Depressed Rats

**FIGURE 4:**
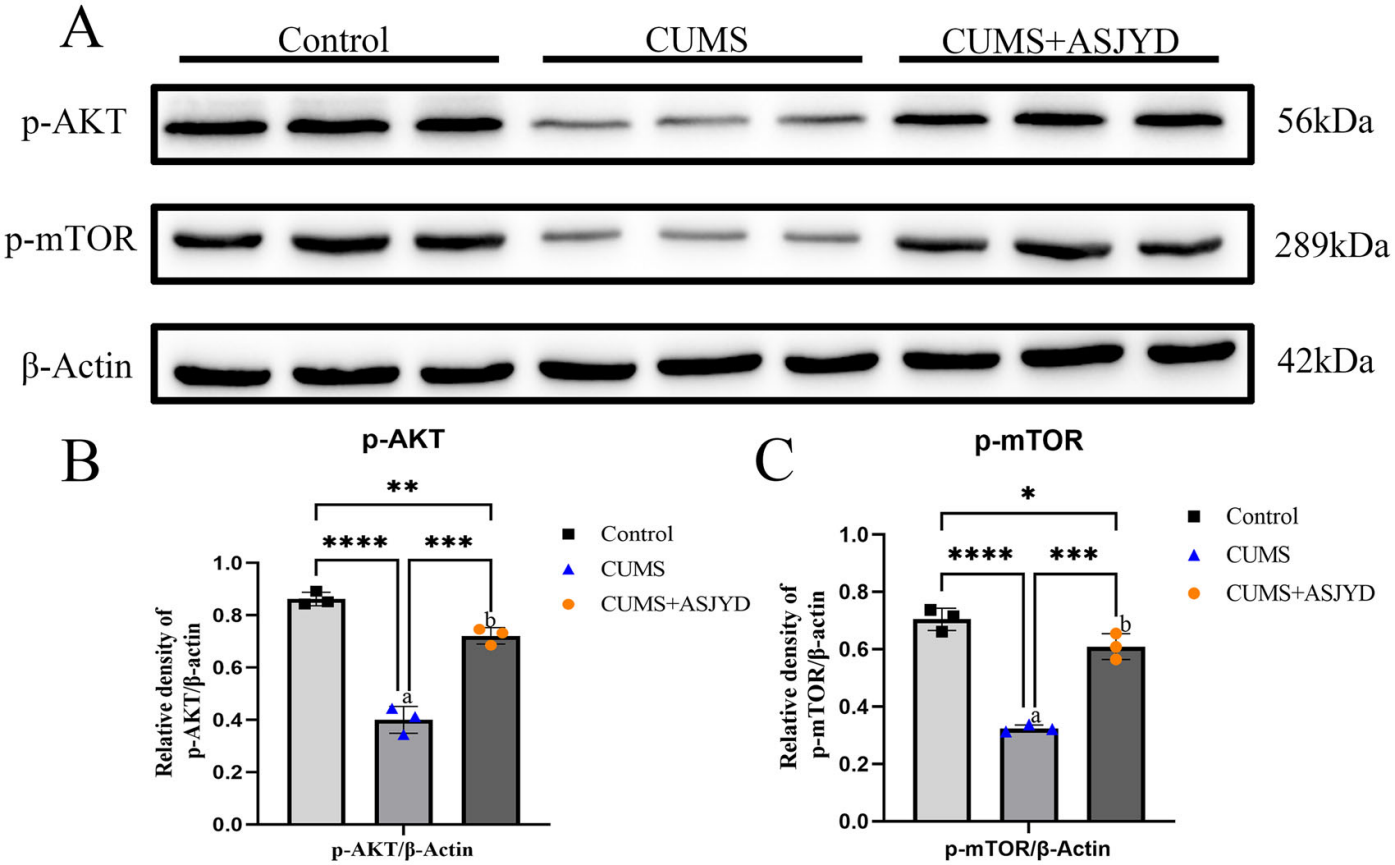
The phosphorylation levels of AKT and mTOR proteins in rat hippocampus. (A) Western blot. (B, C) Quantitative statistical results of the phosphorylation level. ^a^ *p*<0.01 versus Control group, ^b^ *p*<0.01 versus CUMS group. The uncropped/raw Western blot data are provided in File S1 (“WB Raw Data.docx”).

Compared with the Control group, the expression levels of p‐AKT and p‐mTOR in the CUMS group were significantly decreased (*p* < 0.01; Figure 4B,C), indicating reduced phosphorylation of AKT and mTOR under chronic stress. Compared with the CUMS group, p‐AKT and p‐mTOR levels in the CUMS+ASJYD group were significantly increased (*p* < 0.01; Figure 4B,C), suggesting that ASJYD enhances AKT and mTOR phosphorylation in the hippocampus. Total AKT and total mTOR were not assessed; these results are interpreted as changes in phosphorylation levels rather than p/total activation ratios. The uncropped/raw Western blot data are provided in File S1 (“WB Raw Data.docx”).

### 4.5 An Shen Jie Yu Tang Can Improve Hippocampal Tissue Morphology

**FIGURE 5:**
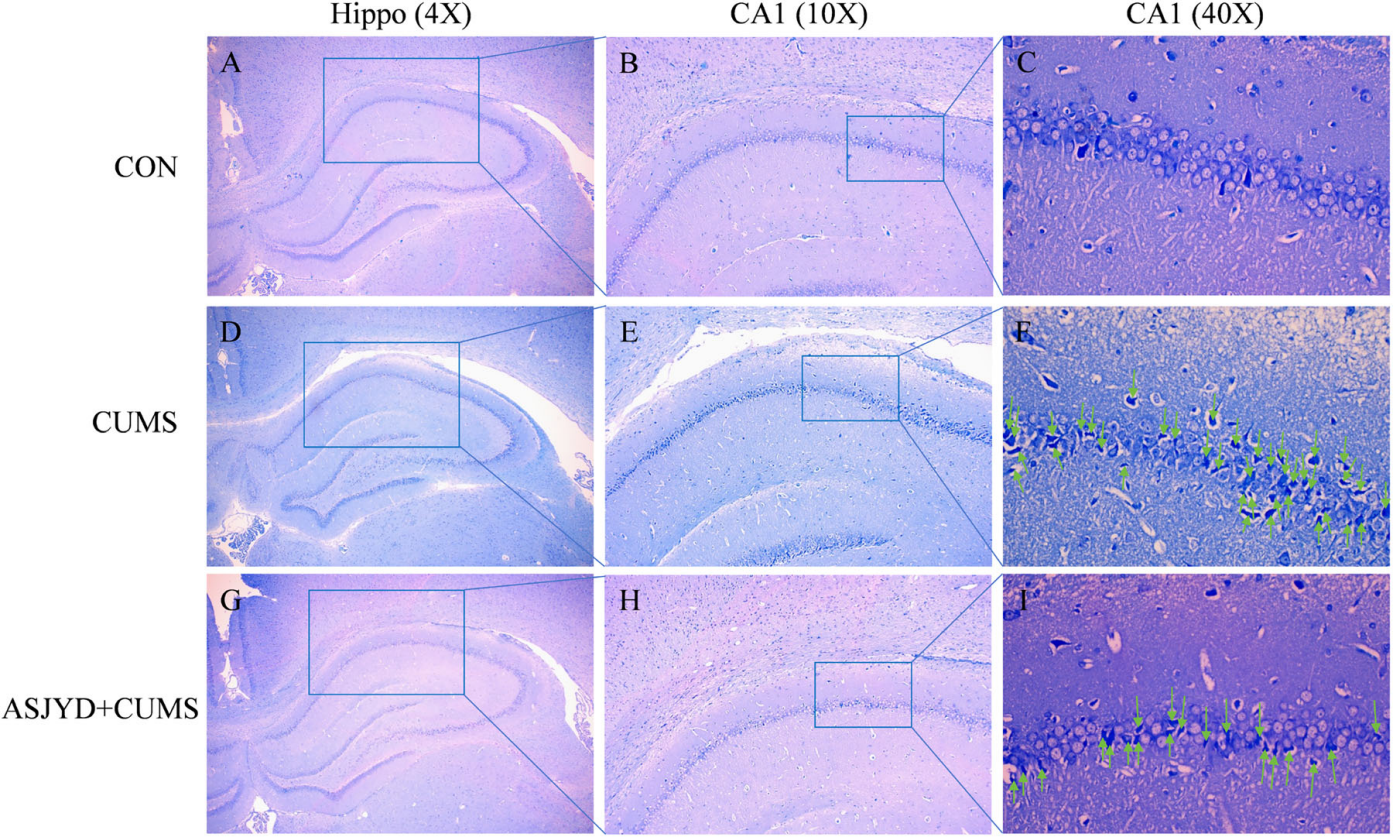
Effects of ASJYD on hippocampal CA1 morphology in rats (Nissl staining). Representative micrographs of the hippocampus/CA1 region from the Control, CUMS, and CUMS+ASJYD groups. Low‐magnification images (4X) and intermediate‐magnification CA1 images (10X) show boxed regions that are displayed at higher magnification (40X) in the corresponding panels. Arrows in the 40X panels indicate representative abnormal neurons in the CA1 region (e.g., pyknotic nuclei and less distinct Nissl staining).

As shown in Figure 5, Nissl staining in the Control group revealed a well‐organized CA1 pyramidal cell layer with neurons showing clear somatic contours and readily identifiable Nissl substance (Figure 5A–C). In the CUMS group, the CA1 pyramidal layer appeared less compact and less organized, with fewer morphologically intact pyramidal neurons observed and more neurons displaying pyknotic nuclei and less distinct Nissl staining (Figure 5D–F). Compared with the CUMS group, the CUMS+ASJYD group showed an apparent improvement in CA1 cytoarchitecture, including a more continuous and orderly pyramidal layer and fewer neurons with pyknotic nuclear morphology (Figure 5G–I). Arrows in the 40× panels indicate representative abnormal CA1 neurons.

### 4.6 Transmission Electron Microscopy Changes in Mitochondrial Morphology

**FIGURE 6:**
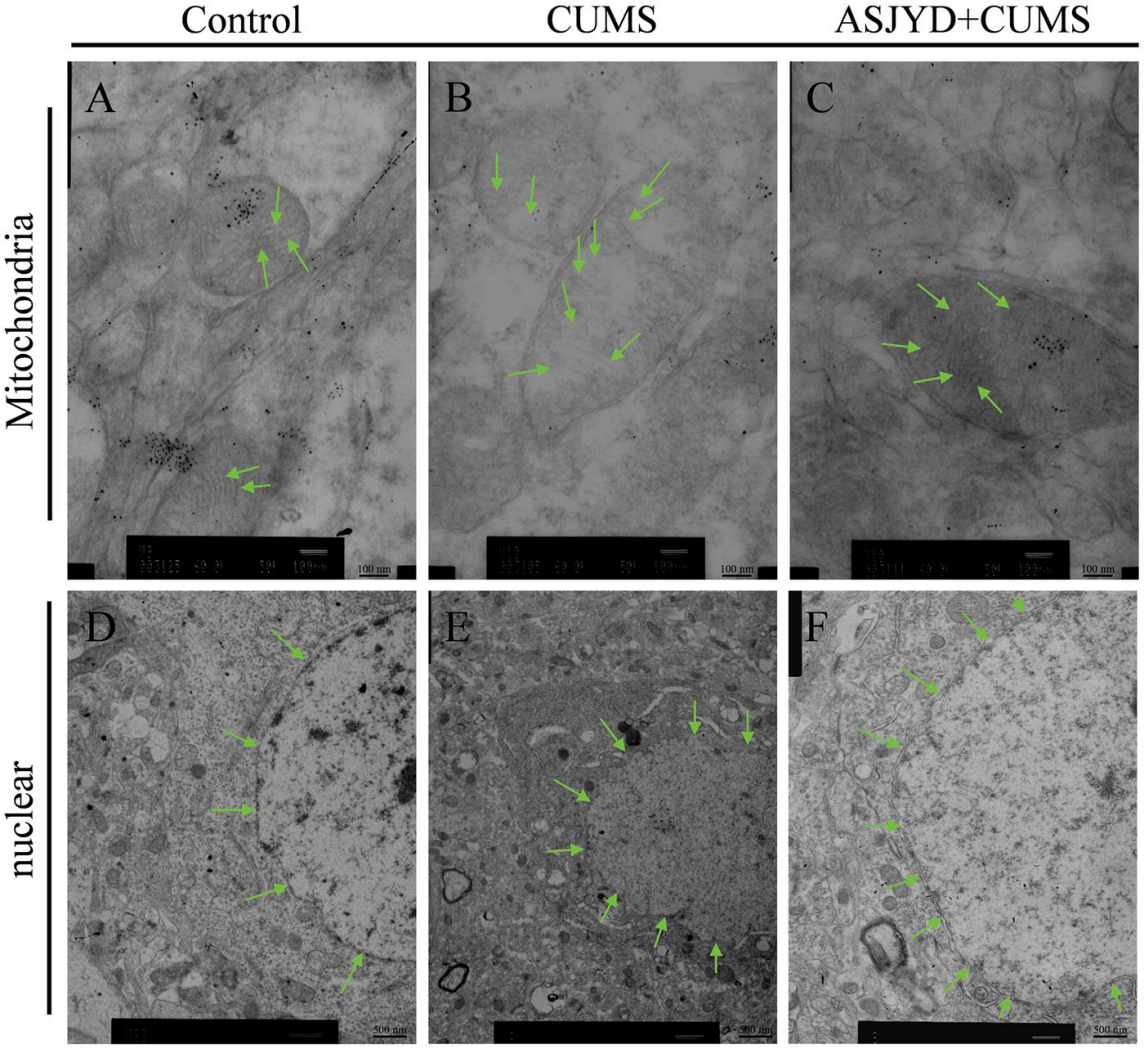
ASJYD ameliorated mitochondrial impairment in the hippocampus of CUMS rats. Bar = 100 nm (A−C) and 500 nm (D−F). A−C (Mitochondria): Representative electron microscopy images of mitochondrial morphology in the Control (A), CUMS (B), and CUMS+ASJYD (C) groups. Arrows indicate key mitochondrial features: intact cristae (Control), mitochondrial swelling and disorganized cristae (CUMS), and improved mitochondrial structure with more organized cristae (CUMS+ASJYD). D−F (Nuclear): Representative electron microscopy images of nuclear morphology in the Control (D), CUMS (E), and CUMS+ASJYD (F) groups. Arrows highlight nuclear contours: intact nuclear membranes (Control), nuclear condensation and disrupted membranes (CUMS), and clearer nuclear boundaries (CUMS+ASJYD).

As shown in Figure 6, TEM revealed clear nuclear structures and intact nuclear membranes in the Control group. Mitochondria appeared oval in shape, with distinct contours, uniform matrix, and densely arranged cristae (Figure 6A,D). In the CUMS group, nuclear condensation and blurred nuclear membrane boundaries were observed. Mitochondria exhibited swelling, matrix loosening, vacuolar‐like changes, and disrupted or fragmented cristae (Figure 6B,E). Compared with the CUMS group, the CUMS+ASJYD group showed apparent morphological improvement. Nuclear membrane boundaries appeared clearer, and mitochondrial morphology showed reduced swelling, relatively preserved matrix integrity, and more organized cristae (Figure 6C–F). These observations are based on representative images and reflect qualitative ultrastructural changes.

## 5 Discussion

The CUMS depression model predominantly involves prolonged exposure to chronic stress, mirroring the extended low mood and depressive states observed in humans. This model is more closely aligned with the pathogenesis and clinical manifestations of depression (Antoniuk et al. 2019; Sharma et al. 2024). In behavioral experiments, rats subjected to the CUMS model demonstrated anhedonia, diminished reward responses, significantly reduced spatial exploration and interest, as well as psychomotor retardation and other pronounced anxiety‐depression‐like behaviors, indicating the successful establishment of the CUMS depression rat model. Post‐treatment, there was a significant amelioration of depressive‐like behaviors in the rats, evidenced by increased sucrose consumption and preference rates. Furthermore, in the OFT, the frequency of exploratory activities by the rats increased substantially, and in the Morris water maze test, the escape latency period was significantly reduced, thereby highlighting the antidepressant effects of the ASJYD formula.

It should be noted that CUMS stress exposure was discontinued during the 14‐day treatment phase. Therefore, the present study evaluates the effects of ASJYD on stress‐induced depressive‐like states after stress cessation rather than pharmacological reversal under continuous stress exposure. This experimental design reflects post‐stress intervention and recovery processes, which should be considered when interpreting the behavioral and molecular findings.

The pathogenesis of depression is characterized by its complexity, involving multifactorial neuroendocrine‐immune mechanisms. Among these, the overactivation of the HPA axis and alterations in monoamine neurotransmitter levels are widely recognized hypotheses (Cui et al. 2024). Within the HPA axis, the hypothalamus secretes corticotropin‐releasing hormone, which stimulates the pituitary gland to release ACTH. ACTH subsequently acts on the adrenal glands to promote the release of CORT, which binds to glucocorticoid receptors (GRs). This process is regulated by a negative feedback mechanism. However, in individuals with depression, prolonged stress stimuli can lead to the downregulation of GR function, resulting in impaired negative feedback regulation (Dwyer et al. 2020). Consequently, excessive release of CORT can cause tissue damage (Gulyaeva 2023). Our experimental findings demonstrated that serum ACTH and CORT levels in the CUMS group of rats were significantly elevated compared to those in the control group, indicating HPA axis overactivation. These results suggest that HPA axis hyperactivity is one of the pathogenic mechanisms underlying depression. Following treatment, there was a significant reduction in serum ACTH and serum CORT levels in rats, accompanied by an improvement in depressive‐like behavior. These findings suggest that ASJYD may partially normalize stress‐induced elevations in serum ACTH and CORT, thereby mitigating symptoms associated with emotional abnormalities and physical discomfort. Sustained glucocorticoid exposure secondary to HPA axis hyperactivation may contribute to downstream mitochondrial dysfunction and alterations in intracellular survival pathways, thereby linking neuroendocrine dysregulation with impaired hippocampal plasticity.

The hippocampus is integral to memory, cognition, and emotional regulation (Katherine L. Jones et al.^2022^). Following the onset of depression, various mechanisms contribute to damage to hippocampal neurons, resulting in abnormal neural connections and functions within this brain region, which subsequently manifest as symptoms such as low mood and diminished interest in learning (Gao et al.^2025^). Moreover, mitochondria, as essential sites for energy production and cellular function maintenance, are pivotal in neural transmission and plasticity within the brain (Chen et al. 2024; Jiang et al. 2024). Research suggests that mitochondrial dysfunction is a characteristic feature of neurodegenerative diseases, with its decline being closely linked to the emergence of psychiatric symptoms. The findings of this experiment indicate that hippocampal neurons in the CUMS group display disorganized arrangements, reduced cell layer density, incomplete nuclei, and nuclear condensation. Following treatment, there was a significant improvement in neuronal pathology, characterized by increased cell layer density, more organized arrangements, and intact nuclei. Electron microscopy revealed that the CUMS group exhibited nuclear condensation, nucleolar dissolution, indistinct nuclear membrane boundaries, severe mitochondrial morphological damage, matrix disorganization, and cristae fractures. In contrast, the ASJYD treatment group demonstrated a marked reduction in structural damage to both hippocampal nuclei and mitochondria, with a well‐organized matrix and regular, intact cristae arrangement. These observations suggest that ASJYD may preserve hippocampal neuronal structural integrity under stress conditions. Given the close relationship between mitochondrial function and intracellular survival signaling, such structural improvements may also be associated with modulation of downstream pathways, including AKT/mTOR signaling.

In the context of stress‐induced HPA axis hyperactivation and mitochondrial alterations observed in this study, the AKT/mTOR signaling pathway may represent a critical downstream node linking neuroendocrine stress responses to cellular survival and neuroplasticity. The AKT/mTOR signaling pathway is ubiquitously expressed across various cell types and has been implicated in depression‐related phenotypes. This pathway may contribute to depression pathophysiology through mechanisms involving hippocampal neuronal survival/apoptosis‐related processes, mitochondrial function, neurotrophic factor signaling, and glutamatergic regulation (Arthur et al. 2023). Numerous growth factors interact with tyrosine kinase receptors, initiating autophosphorylation events that activate PI3K. This activation catalyzes the conversion of PIP2 into PIP3. Subsequent to PIP3 activation, PDK, possessing a pleckstrin homology domain, along with AKT, are recruited to the plasma membrane. AKT undergoes sequential phosphorylation by PDK1 and PDK2 in a PIP3‐dependent manner. Upon activation, AKT can subsequently activate a multitude of downstream signaling proteins through a series of biochemical reactions, thereby exerting a variety of functions (Tang et al. 2024). Among these downstream targets, mTOR plays a crucial role by enhancing hippocampal stem cell function, inhibiting the release of apoptotic factors to prevent neuronal apoptosis, and increasing mitochondrial ATP production, which in turn reduces free radical release and mitigates hippocampal neuronal damage (P. Yang et al. 2023). Phosphorylated AKT and mTOR (p‐AKT and p‐mTOR) are commonly used as phosphorylation‐based indicators of AKT/mTOR signaling changes. Our results showed that p‐AKT and p‐mTOR levels were decreased in the CUMS group. In contrast, p‐AKT and p‐mTOR levels were significantly increased in the ASJYD treatment group (CUMS+ASJYD).

Taken together with the improved hippocampal neuronal morphology after treatment, these findings suggest that ASJYD may exert antidepressant‐like effects, at least in part, by enhancing AKT and mTOR phosphorylation and promoting neuroprotective processes. Because total AKT and total mTOR were not measured, our results should be interpreted as changes in phosphorylation levels rather than definitive pathway activation based on p/total ratios. According to Traditional Chinese Medicine, depression is attributed to emotional disturbances and qi stagnation, and is associated with the heart, liver, and spleen (N. N. Yang et al. 2022). Within the An Shen Jie Yu Tang formula, Bupleurum and Poria are utilized to regulate the liver and alleviate depression, while Longan, Ziziphus, and Polygala are employed for their properties of nourishing the heart, tonifying the liver, calming the mind, and promoting sleep. Additionally, Astragalus and Atractylodes are incorporated to tonify qi and elevate yang.

In conclusion, the findings substantiate that ASJYD ameliorates stress‐induced depressive‐like symptoms in CUMS rats under a post‐stress intervention paradigm. Its antidepressant‐like effects may involve attenuation of stress‐induced HPA axis hyperactivity, preservation of hippocampal mitochondrial and neuronal integrity, and enhancement of AKT/mTOR phosphorylation within a stress–neuroplasticity framework. Additionally, this study elucidates the mechanisms underlying depression. Future investigations should delve into the specific signaling pathways and molecular mechanisms involved. For the treatment of depression, the integration of traditional Chinese and Western medicine, alongside personalized treatment strategies, warrants consideration (Sun et al. 2023).

### 5.1 Limitations

This study has several limitations that should be considered when interpreting the results:

**CUMS model design**:

The CUMS model in this study involved the cessation of stress exposure during the treatment phase, which reflects a post‐stress intervention and recovery model rather than pharmacological reversal under ongoing stress. While this design allows for the exploration of stress‐induced depressive‐like states and the effects of treatment following stress cessation, it may not fully represent the mechanisms of treatment under continuous stress conditions.

**Sample size limitations**:

The sample size used for Western blot analyses (*n* = 3 per group) was relatively small. While this number was chosen based on previous studies of a similar nature, the sample size may limit the generalizability of the findings, and further studies with larger sample sizes are recommended to confirm these results.

**Measurement of total AKT and mTOR**:

Total AKT and mTOR levels were not measured in this study:

The observed effects should be interpreted as changes in phosphorylation rather than definitive pathway activation. Phosphorylation levels were used as functional indicators of AKT/mTOR signaling, and this approach is commonly employed in studies where pathway activation is inferred from phosphorylation data. Future studies may consider measuring total protein levels to further validate the role of AKT/mTOR signaling in depression.

## Author Contributions

**Zhen Li**: Conceptualization, Investigation, Methodology, Supervision, Writing review & editing. **Zijun Ji**: Conceptualization, Formal analysis, Investigation, Writing review & editing. **Zehao Zhang**: Data curation, Formal analysis, Investigation, Writing original draft. **Xuzhang Wang**: Formal analysis, Validation. **Hongyue Yu**: Formal analysis, Validation. **Dongrun Yang**: Formal analysis, Validation. **Huijin Zhang**: Formal analysis, Validation. **Yongsheng Liu**: Conceptualization, Methodology, Software, Validation. **Zhuxin Sui**: Data curation, Funding acquisition, Project administration, Resources, Supervision, Visualization. Zhen Li, Zijun Ji, and Zehao Zhang contributed equally to this work. All authors have read and approved the final manuscript.

## Funding

The present study was funded by the school‐level Research Project of Qilu College of Medicine (grant no. JCYXKFFC202508).

## Conflicts of Interest

The authors declare no conflicts of interest.

## Ethics Approval

Approved by Qilu Medical University's Institutional Review Board, the study protocol carries ethics approval no. YXLL2019001.

## Supporting information




**Supplementary Materials**: brb371380‐sup‐0001‐SuppMatt.docx

## Data Availability

The data that support the findings of this study are available from the corresponding authors upon reasonable request.
