# Supplementary material for: Anshen Jieyu Decoction: A Promising Remedy for Depression via AKT/mTOR Pathway Modulation in CUMS Rats
Source: Brain Behav. 2026 Mar 31;16(4):e71380. doi: 10.1002/brb3.71380 (PMC13112013; doi:10.1002/brb3.71380)
Supplement: Supplementary file 1 — Supplementary Materials: brb371380‐sup‐0001‐SuppMatt.docx [file BRB3-16-e71380-s001.docx]

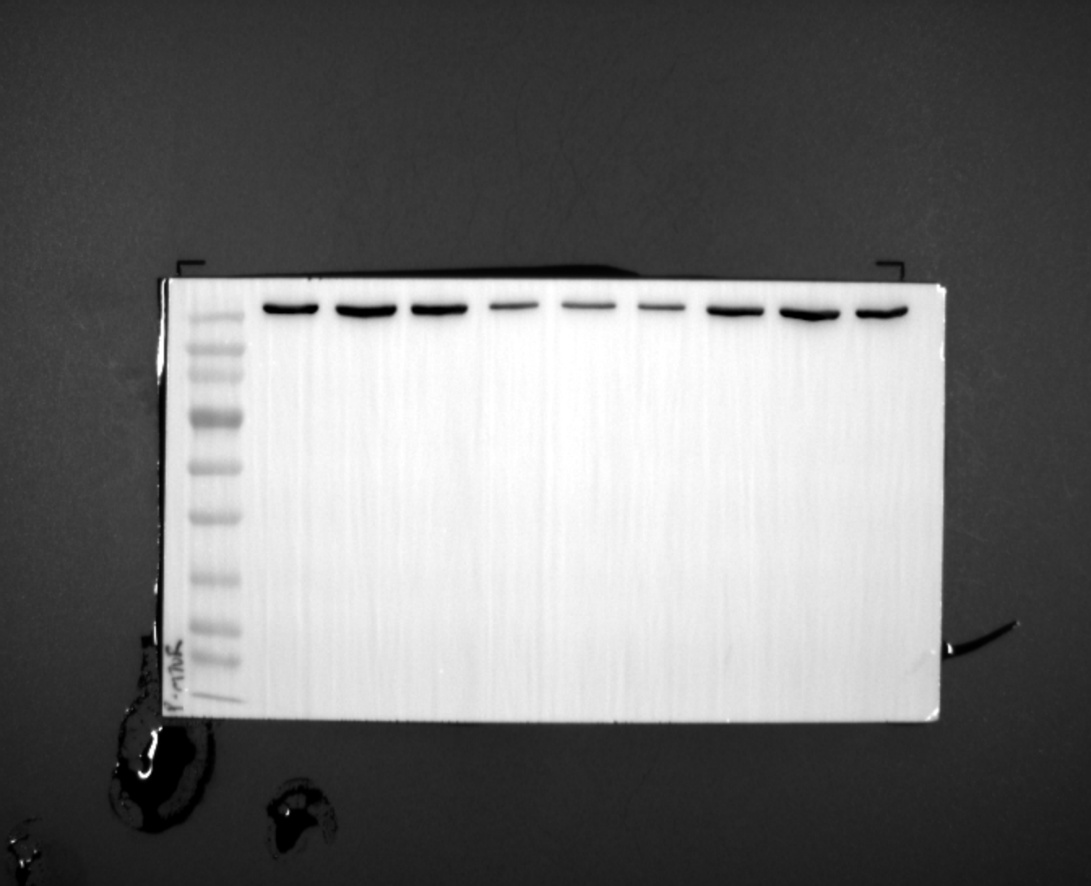

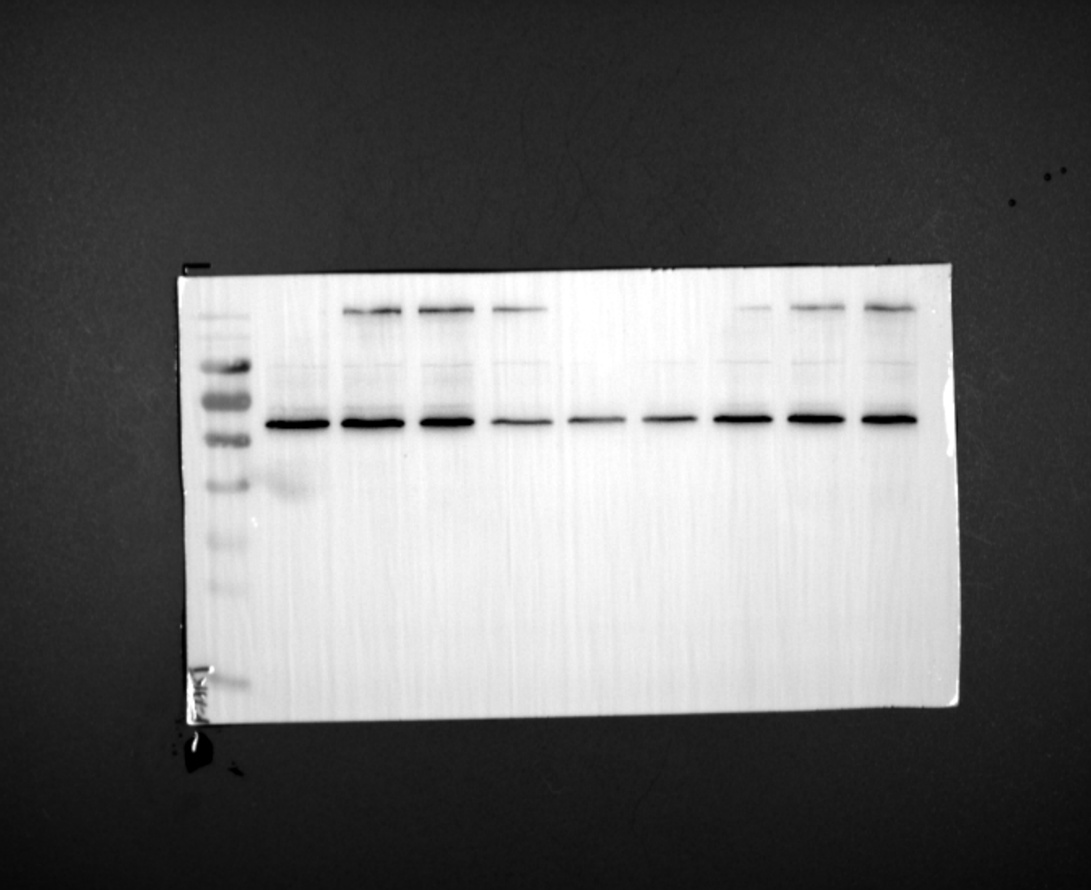

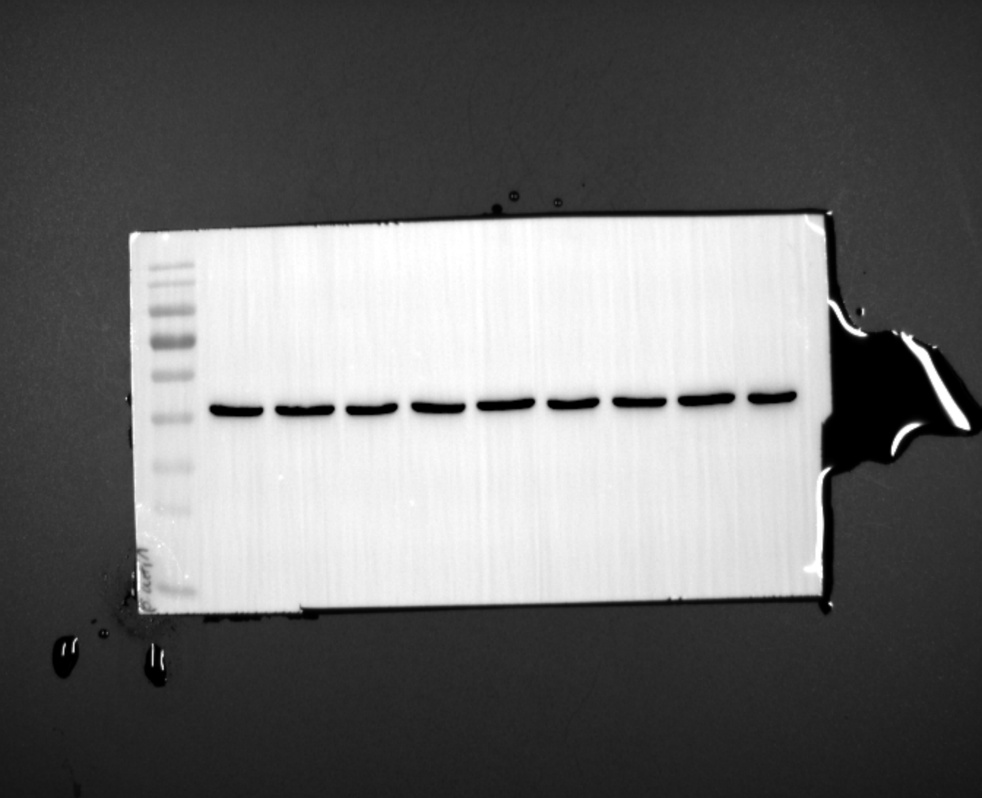


P-AKT (56kDa)

P-mTOR (289kDa)

CUMS+ASJYD

CUMS

Control

CUMS+ASJYD

CUMS

Control

β-Actin (42kDa)

CUMS+ASJYD

Control

CUMS
